# Dopaminergic neurodegeneration in Gerstmann–Sträussler–Scheinker (P102L) disease: insights from imaging and pathological examination

**DOI:** 10.3389/fneur.2024.1452709

**Published:** 2024-09-23

**Authors:** Ken-Ichi Irie, Hiroyuki Honda, Takahisa Tateishi, Shinichiro Mori, Akifumi Yamamoto, Makoto Morimitsu, Kikuchi Shinsuke, Taiga Moritaka, Seiji Kurata, Hiroyuki Kumazoe, Masahiro Shijo, Naokazu Sasagasako, Takayuki Taniwaki

**Affiliations:** ^1^Division of Respirology, Neurology and Rheumatology, Department of Medicine, Kurume University School of Medicine, Kurume, Japan; ^2^Neuropathology Center, NHO Omuta Hospital, Fukuoka, Japan; ^3^Division of Neurology, Department of Neurology, Neuro Muscular Center, NHO Omuta Hospital, Fukuoka, Japan; ^4^Department of Radiology, Kurume University School of Medicine, Kurume, Japan; ^5^Department of Radiology, NHO Omuta Hospital, Fukuoka, Japan; ^6^Department of Neurology, Kyushu Central Hospital of the Mutual Aid Association of Public School Teachers, Fukuoka, Japan

**Keywords:** Gerstmann–Sträussler–Scheinker disease, dopaminergic neurodegeneration, DAT-SPECT, prion protein, P102L mutation

## Abstract

Gerstmann–Sträussler–Scheinker (GSS) disease is an inherited prion disease characterized by dementia, cerebellar ataxia, and painful sensory disturbances. GSS is pathologically defined by the presence of amyloid plaques comprised of prion protein predominantly localized in the cerebral cortex, cerebellar cortex, and basal ganglia, resulting from mutations in the prion protein gene. This study investigated five cases of GSS P102L [GSS caused by a leucine (L) substitution of proline (P) at position 102 of the prion protein gene] with L-dopa-resistant extrapyramidal symptoms and reduced dopamine transporter single-photon emission computed tomography (DAT-SPECT) uptake. Clinical findings revealed diverse manifestations, with all cases exhibiting parkinsonism, and four patients had a vertical gaze palsy. Notably, all patients showed reduced striatal DAT-SPECT uptake, indicating neurodegeneration of the nigrostriatal system. Autopsy findings in one case confirmed prion protein plaques and dopaminergic neuron loss in the substantia nigra of a patient with GSS P102L. Additionally, reduced DAT immunostaining was observed in the putamen compared with a control. While previous studies have identified reduced DAT-SPECT and positron emission tomography uptake in Creutzfeldt-Jakob disease and fatal familial insomnia owing to nigrostriatal neurodegeneration induced by abnormal prion protein deposition, similar phenomena in GSS P102L have not been reported. This study provides support for a correlation between abnormal prion protein deposition and nigrostriatal system degeneration in GSS P102L. Our results reveal the importance of considering GSS P102L in cases of atypical Parkinsonism and abnormal DAT-SPECT results, which would serve as a valuable indicator for subsequent prion genetic testing.

## Introduction

Gerstmann–Sträussler–Scheinker disease (GSS) with the P102L mutation is an autosomal dominant inherited prion disease characterized by cerebellar ataxia, muscle weakness, muscle atrophy, and painful sensory disturbances (1). In advanced stages, cognitive impairment usually complicates the clinical presentation, progressing to akinetic mutism and causing death, typically within an average period of 5–6 years. In Japan, a 10-year prion disease survey since 1999 reported 44 cases of GSS with P102L and P105L mutations; notably, P102L was the predominant mutation, accounting for 89% of cases (2). Progressive supranuclear palsy (PSP)-like symptoms has been reported in GSS (3). Neuroimaging studies in patients with GSS have involved using various techniques, including structural magnetic resonance imaging (MRI), positron emission tomography (PET), single-photon emission computerized tomography (SPECT), and electroencephalography (EEG). While characteristic periodic synchronous discharges (PSD) in EEG are prevalent in approximately 97% of patients with sporadic Creutzfeldt-Jakob disease (CJD) based on surveillance of the Japanese population, individuals with GSS P102L exhibit them less commonly, with an incidence rate of 19% (2). Initial brain MRIs usually do not reveal distinct cerebral and cerebellar atrophy or high-signal lesions on diffusion-weighted imaging (DWI); however, advanced stages exhibit evident cerebral and cerebellar atrophy in most patients, recognized as progressive (4). Notably, a few cases display high-signal lesions in the occipital lobe cortex on DWI several years after onset (5). Laboratory findings in GSS P102L cases included elevated cerebrospinal fluid (CSF) levels of 14–3–3 protein in 27.3% of cases and elevated CSF tau levels in 27.8%. Early diagnosis remains challenging owing to the low incidence of abnormal findings on EEG, brain MRI, and CSF testing.

DAT-SPECT aids in evaluating global functional dopaminergic synaptic changes in the striatum. It helps differentiate between non-degenerative parkinsonisms (e.g., essential tremor, drug-induced parkinsonism) and neurodegenerative parkinsonisms, especially if tremor is present. Degeneration of synaptic neurons and reduced striatal uptake are associated with clinical manifestations of bradykinesia and muscle rigidity in CJD and Parkinson’s disease, parkinsonian disorders (6–8).

Although case reports have linked parkinsonism with DAT-SPECT in sporadic CJD, fatal familial insomnia (FFI), GSS F198S [GSS caused by a serine (S) substitution of phenylalanine (F) at position 198 of the prion protein gene (*PRNP*)], and GSS D202N [GSS caused by a asparagine (N) substitution of aspartic acid (D) at position 202 of the *PRNP*] (9–11), the assessment of dopaminergic function in GSS P102L remains unknown. This study presents five cases of GSS P102L characterized by L-dopa-resistant extrapyramidal symptoms accompanied by reduced uptake observed on DAT-SPECT, demonstrating dopaminergic denervation in GSS P102L through DAT-SPECT and neuropathological investigation. In contrast to reports suggesting that extrapyramidal signs are less prominent in GSS P102L compared to other prion diseases (12), our findings indicate that cases of GSS P102L present with extrapyramidal signs and PSP-like syndrome, albeit to varying degrees, and that these syndromes can be predicted by DAT-SPECT.

## Materials and methods

### Patients and clinical study

The study included five consecutive patients diagnosed with GSS, all of whom tested positive for the P102L genetic mutation in the prion protein gene between April 1, 2020, and March 31, 2023, at Kurume University, Kurume city and National Omuta hospitals, Omuta city in Japan. There was no consanguinity among the patients. Patient information was retrospectively examined through a thorough review of the medical records.

The studies involving human participants were reviewed and approved by Kurume University (research number: 23104) and NHO Omuta National hospital (research number: 5–44). Written informed consent to participate in this study was provided by the next of kin. The detailed clinical features of representative cases are provided below.

### DAT-SPECT

Patients with GSS underwent imaging 3 h after the injection of 167 MBq of ^123^I-ioflupane, specifically [^123^I]-2β-carbomethoxy-3β-(4-iodophenyl)-N-(3-fluorophenyl) nortropane ([^123^I] FP-CIT; Nihon Medi-Physics, Tokyo, Japan). The ^123^I-FP-CIT SPECT images were obtained using a Symbia Evo Excel scanner (Siemens Healthcare). Neurologists performed the initial image evaluation using a visual evaluation method. Subsequently, specific binding ratios (SBRs) in the right and left striata were semi-quantitatively calculated using the DATView software (Nihon Medi-Physics, Tokyo, Japan) based on Bolt’s method, as described in detail elsewhere (13) for Cases 1 to 5.

### Neuropathological study

Postmortem examination of patient 5 was performed 7 h after death. Brain and spinal cord specimens were fixed in buffered 10% formalin for 2 weeks. Following fixation, specimens were immersed in 90% formic acid for 60 min to detoxify PrP, embedded in paraffin, and sectioned into 6-μm-thick slices. Sections were stained with hematoxylin and eosin (HE). For immunohistochemical investigations, the following primary antibodies and dilutions were used: anti-dopamine transporter (rabbit polyclonal antibody, 1:250; Abcam, Cambridge, UK) and anti-8G8 (mouse monoclonal antibody specific for PrP at amino acid residues 95–110, 1:400; Cayman, Ann Arbor, MI, USA) antibodies. Secondary antibodies included horseradish peroxidase-conjugated anti-rabbit (PI-1000) and anti-mouse (PI-2000) (1,200, Vector Laboratories, Burlingame, CA, USA). Immunohistochemistry was performed using an indirect immunoperoxidase method, as previously described. The sections were deparaffinized in xylene, dehydrated in ethanol, and incubated with 0.3% hydrogen peroxide in absolute methanol for 30 min at room temperature to inhibit endogenous peroxidase activity. After rinsing in tap water, the sections were fully immersed in distilled water and heat-treated in 0.01 M citrate buffer (pH 6.0) for the anti-dopamine transporter antibody or in 1.5 × 0.001 mol/L hydrochloric acid for the anti-PrP antibody, using a microwave for 10 min for antigen retrieval. Following this pretreatment, the sections were incubated with a primary antibody diluted in 5% normal goat serum in 20 mM Tris–HCl (pH 7.6) containing 0.5 M NaCl, 0.05% NaN3, and 0.05% Tween 20 (TBST) at 4°C overnight, and subsequently with a 1:200 dilution of the appropriate secondary antibody for 1 h at room temperature. A colored reaction product was developed using 3, 3′-diaminobenzidine tetrahydrochloride (DAB, Dojindo, Kumamoto, Japan) solution (0.02% DAB, 0.003% H2O2, 50 mM Tris–HCl, pH 7.6). The color development time was set to 180 s. The sections were lightly counterstained with hematoxylin. Duchenne muscular dystrophy was used as a control for the GSS.

### Case series

#### Case 1

**Figure 1:**
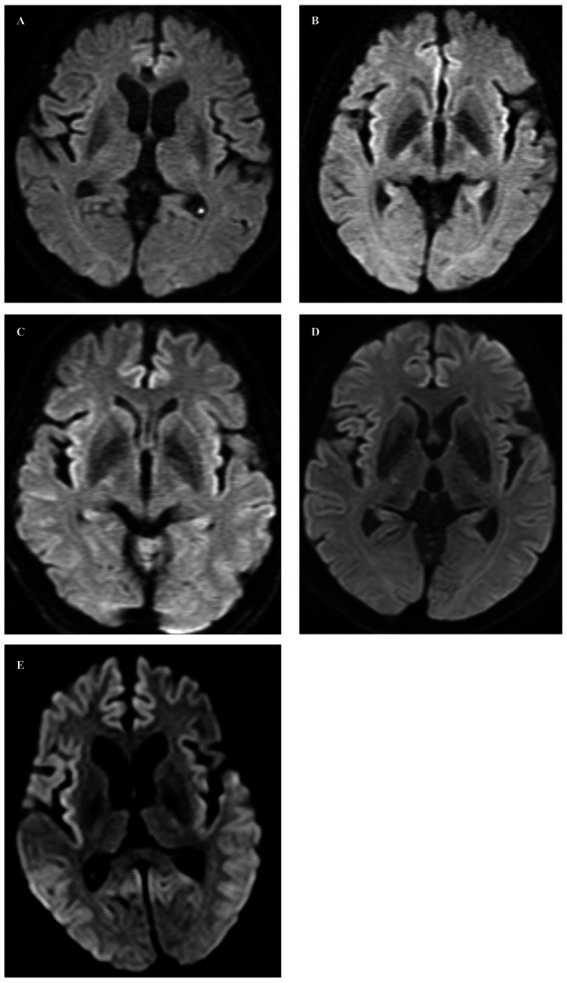
In axial diffusion-weighted images, no abnormal signals were observed in Cases 1 through 4 **(A–D)**, whereas high signal intensity lesions were observed in the cortex of the frontal, temporal, and occipital lobes on both sides, and ADC low density (not shown) corresponding to the high signal intensity was observed in Case 5 **(E)**.

**Figure 2:**
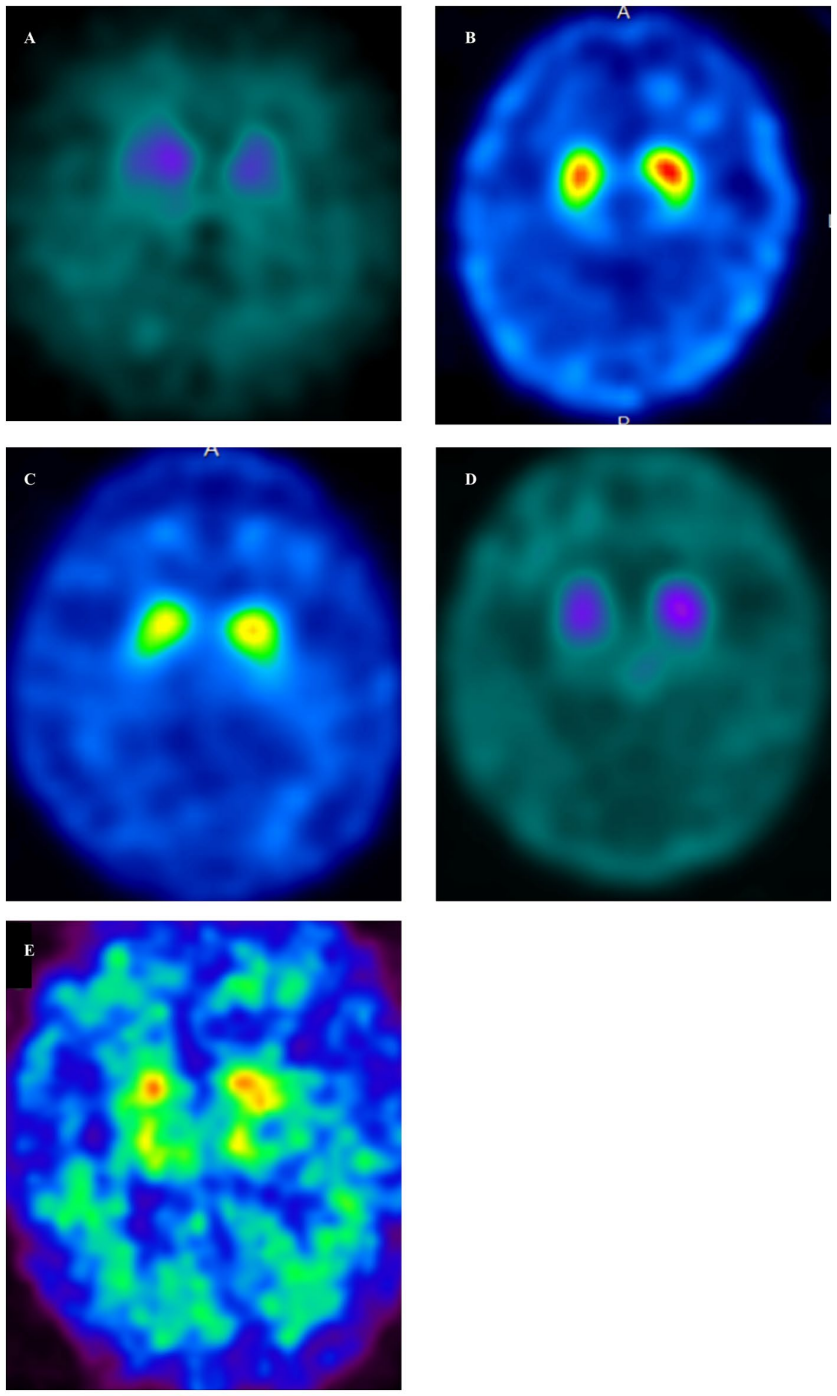
Dopamine transporter single-photon emission computed tomography (DAT-SPECT) (123I-FP-CIT) in GSS cases **(A–E)**. DAT-SPECT demonstrated decreased uptake in the bilateral putamen in all cases. (**A**, Case 1; **B**, Case 2; **C**, Case 3; **D**, Case 4; **E**, Case 5). **(A)** There was an overall decrease in uptake, accompanied by changes in the shape of the striatum on both sides. Specific binding ratios (SBRs) were 1.29 (left) and 2.07 (right). **(B)** There was a decreased uptake in the posterior part of both putamen, along with a dot-like accumulation. The SBRs were 3.85 (left) and 4.08 (right). **(C)** Decreased uptake was demonstrated in the posterior part of both putamen, accompanied by a dot-like accumulation. The SBRs were 3.84 (left) and 3.66 (right). **(D)** There was an overall decrease in uptake, accompanied by changes in the shape of the striatum on both sides. The SBRs were 1.55 (left) and 1.86 (right). **(E)** There was an overall decrease in uptake, accompanied by changes in the shape of the striatum on both sides. The SBR are 1.12 (left) and 1.05 (right).

Case 1 involved a 69-year-old male who, 2 years before his visit, experienced dizziness, slowness of movement, gait disturbance, and abnormal sensations in both lower extremities. He had a family history of spinocerebellar degeneration, with his father and his first cousins (his aunt’s sun and daughter). Despite oral administration of L-dopa, his symptoms persisted, prompting him to seek medical attention. General medical examinations revealed no abnormalities. Cognitive function screening tests showed a mini-mental state examination (MMSE) score of 29/30 (normal range > 23) and a frontal assessment battery (FAB) score of 16/18 [with 12/18 being the threshold that reasonably distinguishes between frontotemporal dementia and Alzheimer’s disease (14)]. Neurological examination revealed no ptosis and severe downward vertical (supranuclear) gaze palsy. While facial sensation and muscles were normal, dysarthria was observed. The movements were slow and bradykinetic, with moderate and mild muscle rigidity observed from the neck to the trunk and in the limbs, respectively. Limb tremors were absent, and limb strength was normal. Impairments were noted in the finger-tapping test, diadochokinesis, and lower extremity agility. The finger-nose test showed mild dysmetria. Deep tendon reflexes were absent in all extremities, and no bilateral pathological reflexes were observed. Although the patient could walk, tandem gait was challenging, and retropulsion was not observed. No bladder or bowel disturbances were observed. Laboratory tests, including complete blood count, creatinine kinase levels, liver enzyme levels, and fasting blood sugar (120 mg/dL), were within normal limits, except for a slightly elevated HbA1c to 6.8%. CSF examination revealed normal results (protein, 45 mg/dL; cell count, 1 cell/μL). T1-weighted MRI revealed moderate diffuse cerebral atrophy with no evidence of midbrain tegmentum atrophy or the hummingbird sign. DWI revealed no areas of hyperintensity in the cerebral cortex, striatum, or thalamus (Figure 1A). DAT-SPECT revealed reduced accumulation in the bilateral striatum, with a specific binding ratio (SBR) of 2.07 on the right side and 1.29 on the left side, with an average of 1.68 and an asymmetry index of 46.9% (Figure 2A). However, 99mTc-ECD SPECT did not reveal cerebellar hypoperfusion. Metaiodobenzylguanidine myocardial scintigraphy revealed a decreased cardiac-mediastinum ratio of 1.44 in the early phase and 1.33 in the late phase. EEG did not show PSD, and peripheral nerve conduction studies revealed no abnormalities in the upper or lower extremities. CSF prion disease-related protein measurements indicated negative results for total tau protein (676 pg/mL, normative range, 1,300 pg/mL), 14–3–3 protein via a semi-quantitative method, and real-time quaking-induced conversion (RT-QUIC) method. Genetic analysis using genome sequencing, revealed a mutation from Pro to Leu in codon 102, Met/Met type at codon 129, confirming GSS diagnosis. According to the clinical phenotypes of GSS P102L described by Tesar et al. he exhibited GSS with areflexia and paresthesia (15). During hospitalization, the L-dopa dose was increased from 150 to 300 mg/day, causing a slight gait improvement. However, a year after his admission, his cognitive function and psychiatric symptoms deteriorated, leading to his transfer to a psychiatric hospital.

#### Case 2

Case 2 involved a 63-year-old female who experienced abnormal sensations and pain in both lower extremities. She developed motivation and memory loss, prompting her to consult a doctor 1 year before. The patient exhibited moderate cognitive dysfunction, including recent memory impairment and attention deficit. She predominantly experienced slowness of movement and paresthesias in the lower extremities, prompting a referral to our hospital. Neurological examination revealed reduced visuospatial cognition and attention and memory deficits. The patient exhibited vertical supranuclear gaze palsy, saccadic eye movements, and dysarthria. Similarly, she experienced bradykinesia, cerebellar ataxia, and painful paresthesias in both lower extremities. Deep tendon reflexes were absent in the lower extremities. Blood count and biochemistry were normal, while CSF analysis showed a normal cell count of 1/μL and an increased protein level of 71 mg/dL. CSF prion disease-related protein measurements indicated negative results for 14–3–3 protein using both the semi-quantitative method and RT-QUIC method. Brain MRI showed no significant cerebral atrophy on T1-weighted images, and DWI showed no hyperintensity in the cerebral cortex, striatum, or thalamus (Figure 1B). DAT-SPECT revealed decreased uptake in the bilateral striatum, with SBRs of 4.08 and 3.85 on the right and left sides, respectively, averaging 3.96, and an asymmetry index of 5.6% (Figure 2B). EEG did not indicate PSD, and peripheral nerve conduction studies showed no abnormalities in the upper or lower extremities. Genetic analysis revealed a mutation from Pro to Leu in codon 102, Met/Met type at codon 129, confirming a GSS diagnosis. She had a family history of the same GSS P102L mutation in her younger brother. Her clinical phenotype of GSS P102L, as described by Tesar et al. was GSS with areflexia and paresthesia (15). One year after her admission, she was using a wheelchair, and 2 years later, she became bedridden.

#### Case 3

Case 3 involved a 65-year-old male patient. A year before his hospital visit, he experienced dizziness while walking, prompting him to consult a doctor who found no abnormalities on the brain MRI. Subsequently, the patient was referred to our hospital. At presentation, he exhibited normal cognitive function, vertical supranuclear gaze palsy, dysarthria, normal muscle strength, mild bradykinesia in the extremities, cerebellar ataxia in the extremities and trunk, and abnormal sensation in both lower extremities. Deep tendon reflexes were absent in both lower extremities. Blood count and biochemistry were normal, and the CSF test showed a cell count of 1/μL and protein levels of 40 mg/dL. CSF prion disease-related protein measurements indicated a negative result for 14–3–3 protein via a semi-quantitative method and the RT-QUIC method. DWI of the brain MRI did not reveal hyperintensity (Figure 1C), while T1-weighted imaging showed mild bilateral frontal lobe atrophy and no atrophy in the brainstem or cerebellum. DAT-SPECT indicated decreased uptake in the putamen region of the bilateral striatum, with SBRs of 3.49 and 3.84 on the right and the left sides, respectively, averaging 3.66, and an asymmetry value of 9.4% (Figure 2C). EEG did not reveal PSD. Genetic analysis revealed a mutation from Pro to Leu at codon 102, Met/Met type at codon 129, prompting a GSS diagnosis. His clinical phenotype of GSS P102L, as described by Tesar et al. was GSS with areflexia and paresthesia (15).

#### Case 4

Case 4 involved a 58-year-old woman who, 1 year before her visit, began experiencing abnormal sensations in both lower limbs, unsteadiness when walking, and frequent falls, prompting a referral by her local neurosurgeon. Her father had difficulty walking due to an unknown degenerative disease. During her visit, her cognitive function was normal, and muscle strength was within the normal range. However, she exhibited abnormal sensation in both lower extremities, limb and trunk ataxia, along with trunk-dominant rigidity. Deep tendon reflexes were absent in both lower extremities. Blood tests yielded normal results, and CSF analysis indicated a cell count of 1/μL and protein levels of 52 mg/dL. CSF analysis for prion disease-related protein indicated a negative result for 14–3–3 protein, as assessed by semi-quantitative method, and the RT-QUIC method. DWI of brain MRI did not reveal hyperintensities (Figure 1D), whereas T1-weighted imaging showed mild bilateral frontal lobe atrophy, with no atrophy observed in the brainstem or cerebellum. IMP-SPECT demonstrated decreased blood flow in the bilateral thalamus; however, no such reduction in blood flow was observed in the cerebellum. DAT-SPECT revealed an overall decrease in uptake, accompanied by changes in the striatum shape on both sides, with SBRs of 1.86 and 1.55 on the right and left sides, respectively, averaging 1.71, and an asymmetry value of 18.1% (Figure 2D). Genetic analysis revealed a mutation from Pro to Leu at codon 102, Met/Met type at codon 129, prompting a GSS diagnosis. Her clinical phenotype of GSS P102L, as described by Tesar et al. was consistent with GSS with areflexia and parethesia (15). She was using a wheelchair 2 years after her admission.

#### Case 5

Case 5 involved a 78-year-old woman who developed a gait disorder 9 years before her visit. Similarly, 5 years before her visit, she had experienced abnormal sensations in both lower limbs and dysarthria. Furthermore, 3 years before her visit, she experienced frequent falls and bradykinesia. At the time of her visit, she presented with a vertical eye movement disorder, truncal ataxia, trunk-dominant rigidity, and abnormal sensations in both lower limbs. Deep tendon reflexes were absent in both lower extremities. The blood tests yielded normal results. CSF prion disease-related protein measurements indicated a negative result for 14–3–3 protein via the semi-quantitative method. DWI of the brain revealed hyperintensity within the cortical ribbon of the frontal, temporal, and parietal lobes (Figure 1E), and atrophy in the cerebellum on FLAIR imaging. Her EEG showed PSD, raising the suspicion of prion disease. DAT-SPECT indicated Decreased uptake in the putamen region of the bilateral striatum with SBRs of 1.05 and 1.12 on the right and left sides, respectively, with an average of 1.09 and an asymmetry index of 6.5% (Figure 2E). Genetic analysis revealed a mutation from Pro to Leu at codon 102, Met/Met type at codon 129, prompting GSS diagnosis. She had a family history of the same GSS P102L mutation in her younger brother. Her clinical phenotype of GSS P102L, as described by Tesar et al. was GSS with areflexia and paresthesia (15). The patient died 4 years after hospitalization, and an autopsy was performed.

## Results

### Neuropathological findings in Case 5

The brain weighed 1,020 g and manifested diffuse moderate atrophy. Cross-sectional examination revealed severe atrophy in the cortex and white matter of the cerebrum, with discoloration and degeneration observed in the basal ganglia (predominantly in the globus pallidus) and lateral thalamus. Similarly, marked atrophy was observed in the cortex and dentate nucleus of the cerebellum. Histologically, the cerebral cortex showed severe spongy changes in the neuropil, along with a severe loss of pyramidal cells with atrophy and the presence of numerous PrP plaques and reactive astrocytes (Table 1).

**Table 1:** Clinical summary of five patients with GSS P102L.

Case	Age at onset, y/sex	Duration from onset (y)	Family history	Ataxia	Supranuclear palsy	Extrapyramidal sign	Areflexia of lower limbs	Dysesthesia in lower limbs	Brain magnetic resonance imaging abnormalities	Electroencephalography periodic synchronous discharge findings	DAT-SPECT	Nerve conduction studies	Prion gene mutation P102L	Codon 129	14–3–3 protein	RT-QUIC
1	69/M	2	+	+	+	+	+	+	−	−	+	+	+	Met/Met	−	−
2	63/F	4	+	+	+	+	+	+	−	−	+	+	+	Met/Met	−	−
3	65/M	1	−	+	+	+	+	+	−	−	+	N/A	+	Met/Met	−	−
4	58/F	1	+	+	−	+	+	+	−	−	+	+	+	Met/Met	−	−
5	78/F	9	+	+	+	+	+	+	+	+	+	+	+	Met/Met	+	N/A

Ataxia included dysarthria, trunk ataxia, and limb ataxia.

Extrapyramidal Sign included hypokinesia, akinesia, bradykinesia, and rigidity.

Nerve conduction study included decreases in compound muscle action potential (CMAP) and sensory nerve action potential (SNAP), prolonged distal latency, and prolonged F-wave latency.

N/A, not available; +, positive; −, negative.

**Figure 3:**
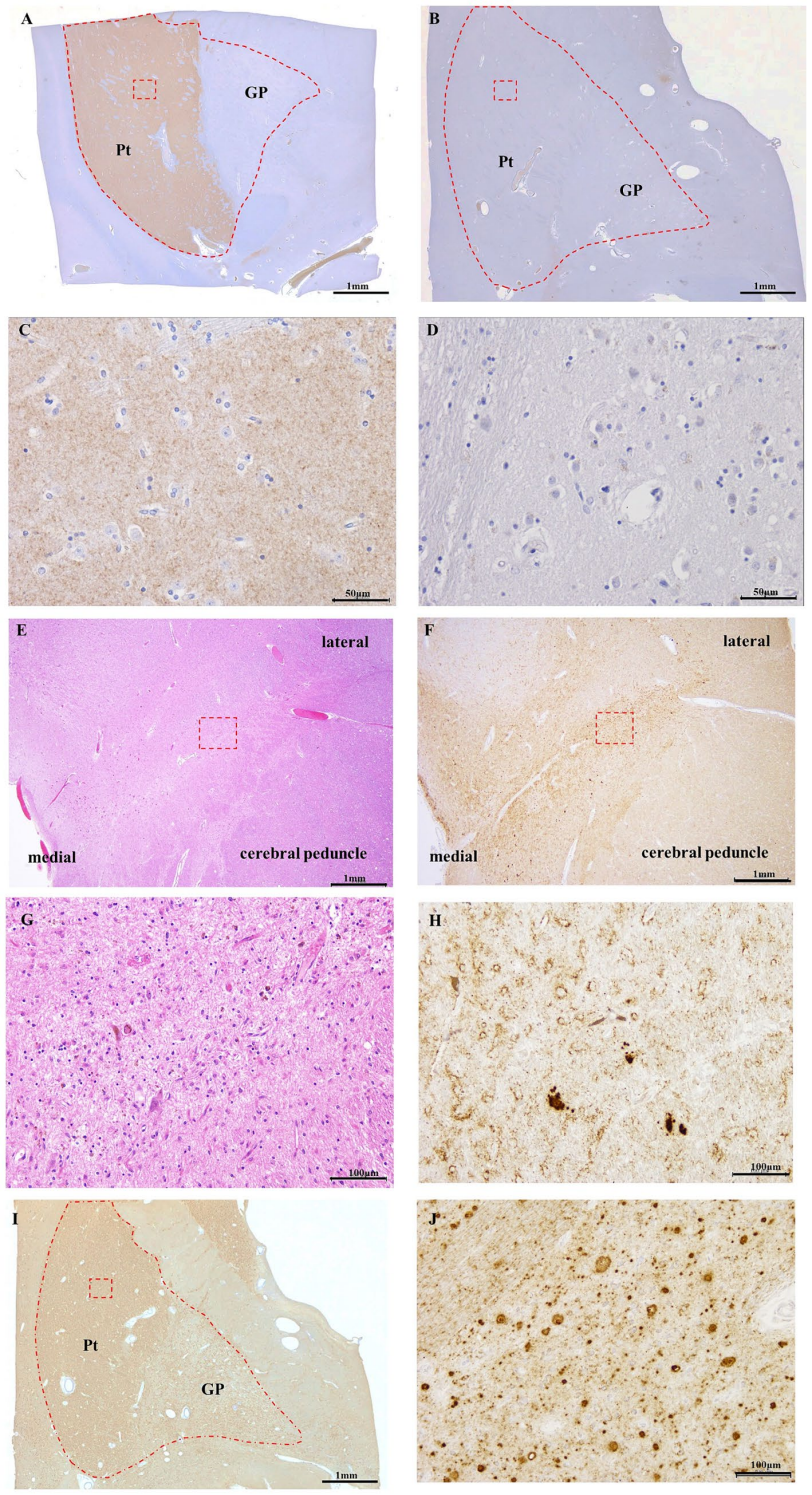
DAT immunostaining of control **(A,C)** and Case 5 **(B,D)**, 8G8 immunostaining of Case 5 **(F,H–J)**, HE staining of Case 5 **(E,G)**. Panels **(C,D,G,H,J)** are high-magnification images of the red square area in Panels **(A,B,E,F,I)**, respectively. Semi-macro images of DAT immunostaining in GSS cases **(B)** and controls **(A)** DAT immunoreactivity is evident in the putamen of the control case. **(B)** DAT immunoreactivity was absent in the putamen of Case 5. DAT immunoreactivity was present in the neuropil of the putamen in the control case **(C)**, but there was no immunopositivity for DAT in the putamen in Case 5 **(D)**. In the 8G8 immunohistochemistry of the putamen in Case 5, PrP immunoreactivity was observed in the midbrain **(F)** and putamen **(I)**, and PrP plaques were markedly observed **(H,J)**. HE staining reveals neuronal loss in the substantia nigra **(E,G)**. *Scale bars:* 1 mm **(A)**, 1 mm **(B)**, 50 μm **(C)**, 50 μm **(D)**, 1 mm **(E)**, 1 mm **(F)**, 100 μm **(G)**, 100 μm **(H)**, 1 mm **(I)**, and 100 μm **(J)**.

In the control group, DAT immunostaining was evident in the putamen of the basal ganglia (enclosed within the dashed line); however, no DAT staining was observed in the globus pallidus (Figure 3A). In contrast, Case 5 exhibited no DAT staining in the putamen or globus pallidus (Figure 3B). Furthermore, under high magnification, DAT staining in the neuropil within the putamen was observed in control cases (Figure 3C). Conversely, Case 5 lacked DAT staining in the neuropils (Figure 3D). HE staining of the midbrain substantia nigra indicated neuronal loss (Figures 3E,G). Additionally, 8G8 immunostaining revealed prion protein deposits in the substantia nigra pars compacta (Figures 3F,H). Immunostaining using the 8G8 antibody in Case 5 demonstrated positive staining in the putamen and globus pallidus, with pronounced PrP plaques observed in the putamen at high magnification (Figures 3I,J).

## Discussion

This cases study of five patients with GSS P102L demonstrates that all patients exhibit parkinsonism, with one showing prominent parkinsonism (Case 1) and the remainder presenting with mild parkinsonism (Cases 2–5). Four patients (Cases 1,2,3, and 5) exhibited supranuclear gaze palsy. Reduced uptake in both striata was observed in all cases involving DAT-SPECT. Additionally, in one autopsy case, the putamen exhibited Decreased immunostaining for DAT in addition to prion protein deposition. This is the first study to reveal dopaminergic denervation in the striata of patients with GSS P102L using DAT-SPECT and neuropathological examination.

GSS, a rare genetic prion disease, presents a diagnostic challenge because of its low incidence (1–100 per 100 million people per year) (16) and many symptoms, including cognitive decline, mental disorders, pyramidal signs, extrapyramidal signs, cerebellar ataxia, and lower limb sensory impairment, with onset ages ranging from 30 to 60 years. Symptom variability and lack of family history further complicate early diagnosis (17–19). In addition, the following abnormal findings are specific to prion diseases, but their sensitivity is low in both MRI imaging abnormalities (9–29%) at early disease stages (4, 20), EEG abnormalities (0–13.5%) (4, 20, 21), and positive CSF14-3-3 protein (18%) (4). RT-QUIC assay has significantly improved diagnostic accuracy due to its high sensitivity and specificity (64–90%)(18, 22). Nevertheless, confirming mutations in the prion protein gene is crucial, particularly in cases without a family history or with unknown family history of the GSS P102L mutation (4), making early diagnosis more complex. The slowly progressive symptoms such as cerebellar ataxia, pyramidal and extrapyramidal signs, and mental disorders can lead to misdiagnosis of PSP, spinocerebellar degeneration, or multiple sclerosis owing to the absence of effective early-stage biomarkers and imaging tests (22–26).

Dopamine released into the nerve terminals of nigrostriatal neurons is taken up by dopamine transporters within nerve terminals, terminating nerve transmission. In addition, dopamine reuptake into synaptic vesicles is stored and recycled. DAT-SPECT was used for the visual evaluation of the dopamine transporter.

DAT-SPECT is used to assess dopamine transporter function and diagnose parkinsonism by visualizing dopamine reuptake in dopaminergic neuron terminals (27). While GSS P102L has not been previously reported in relation to DAT-SPECT, similar abnormalities have been noted in other prion diseases like FFI, CJD, and GSS with other mutations. Abnormal tracer uptake in the presynaptic dopaminergic system has also been observed in other rare diseases such as fragile X-associated tremor/ataxia syndrome, Huntington’s disease, spinocerebellar ataxia, hereditary spastic paraparesis, metabolic disorders, anti-IgLON5 disease, ring chromosome 20 syndrome, chorea-acanthocytosis, and neuronal ceroid lipofuscinosis (28). Patients with FFI develop parkinsonism, dementia, and visual hallucinations, with normal DWI of MRI and DAT-SPECT showing Decreased bilateral striatum uptake (29). A report on parkinsonism caused by sporadic fatal insomnia, a CJD subtype, 2-[18F] Fluoro-2-deoxy-D-glucose PET ([18F] FDG-PET), revealed decreased putaminal accumulation (30). Several CJD cases exhibit parkinsonism, and PET/SPECT reports have indicated Decreased putamen uptake. In prion disease-associated parkinsonism, DAT-SPECT may offer higher sensitivity (7, 8, 31). Vital et al.’s studies on CJD with parkinsonism confirmed that prion protein deposition in the putamen, caudate nucleus, and substantia nigra is associated with presynaptic dopaminergic neuron damage (32). Nuclear medicine examinations with 18F FDG-PET in patients with concurrent CJD and parkinsonism by Xing et al. showed decreased dopamine metabolism in the striatum and thalamus (33). Ufkes et al. reported a case of GSS P102L with PSP-like symptoms, such as parkinsonism, akinesia, ataxia, horizontal and vertical saccadic eye movements, and supranuclear vertical eye movement disorder. Although their case suggested nigrostriatal dysfunction in GSS, this was not confirmed through nuclear medicine or pathological findings (3). This case presented with symptoms similar to cerebellar ataxia predominant PSP (PSP-C) but progressed more rapidly, within one and a half years compared to the typical progression of 4–5 years for PSP-C (34). Costanzo et al. noted that polymorphism at codon 129 in CJD may lead to PSP-like syndrome (35). In our study, four cases with PSP-like syndrome were Met/Met at codon 129, while the case reported by Stephen et al. with Met/Val predominantly exhibited slowly progressive cerebellar ataxia. This suggests that the genotype at codon 129 might influence the presentation of PSP-like syndrome in GSS (4). Although these were consecutive cases, it is highly likely that the same genotype at codon 129 was responsible for the rare PSP-like syndrome in GSS.

Normal prion protein functions as a neuronal membrane protein, regulating the intracellular transport and localization of its binding partners (36). It is highly expressed in the striatum, hippocampus, and prefrontal cortex as a cell-surface glycosylphosphatidylinositol-anchored protein (37) and is distributed in cells within the presynaptic and postsynaptic regions (38). Studies in mice have indicated the presence of intracellular prion proteins in the striatum within synapses, including dopaminergic neurons and receptors, which is crucial in dopaminergic system homeostasis (39). Additional studies in mice have shown that the deposition of abnormal prion proteins in presynaptic dopaminergic neurons impairs the release of synaptic vesicles at nerve terminals and synaptic transmission (40).

We propose that the observed reduction in DAT-SPECT accumulation in our study and the decreased DAT staining in the putamen of an autopsy case result from abnormal prion protein deposition in the presynaptic region, causing nerve terminal damage. Pathologically, we observed the presence of prion protein deposition and loss of dopaminergic neurons in the substantia nigra of an individual with GSS P102L. One could hypothesize that the propagation of abnormal prion proteins through synapses via exocytosis (41) may have contributed to the deposition of abnormal prions in the postsynaptic region, potentially explaining the observed L-dopa refractoriness in Case 1.

Our cases, in which DAT-SPECT was performed 1–4 years after GSS onset, showed abnormal findings, suggesting its utility as an early diagnostic tool from as early as 1 year post-onset.

This study had some limitations. Firstly, it was confined to only two facilities, leading to a small number of registered patients, which limited the ability to fully characterize GSS P102L. Secondly, pathology confirmation was achieved in only one patient. It is essential to assess whether the imaging characteristics obtained through DAT-SPECT align with pathological findings across a larger number of cases. While the PrP plaque deposition in the basal ganglia is commonly reported in pathological studies of GSS, the density of these plaques may influence the clinical manifestation of extrapyramidal symptoms. However, as this study is based on a single case, further pathological investigations involving more GSS cases are necessary (42). In addition, imaging data of DAT-SPECT have been confirmed not only in Parkinson’s disease but also in degenerative diseases such as spinocerebellar degeneration (43). It indicates that many patients with various genetic disorders, especially those with parkinsonism, can exhibit abnormal DAT-SPECT findings. It is necessary to recognize that DAT-SPECT findings in this study are not specific but rather suggestive of neural degeneration that could result from prion protein deposition.

## Conclusion

Our study indicates that PSP-like presentations can occur in GSS and should be considered in the differential diagnosis during the early stages of the disease. We suggest that DAT-SPECT can show abnormalities in patients with GSS who present with extrapyramidal symptoms or vertical gaze palsy. Thus, although GSS is a rare condition, it should be considered in the differential diagnosis of patients with an abnormal DAT-SPECT scan. This consideration should prompt inquiries about family history and the possibility of genetic testing for GSS.

Additionally, this study supports previous findings that PrP plaques are localized to the basal ganglia in GSS. In our case, we found PrP plaques were associated neuronal loss and reduced DAT staining in the putamen. These pathological findings correlate with the clinical features observed in this case series, such as extrapyramidal symptoms or vertical gaze palsy. The observation that four cases presented with vertical gaze palsy and five cases exhibited areflexia and paresthesia, according to the classification by Teser et al. and that the GSS cases occurred at a relatively late age, suggests these factors, in addition to the genotype at codon 129, might reflect characteristics that predict our findings.

Further investigation is necessary to elucidate the relationship between prion protein accumulation, degeneration of the nigrostriatal system, and genetic abnormalities in GSS.

## Data Availability

The original contributions presented in the study are included in the article/supplementary material, further inquiries can be directed to the corresponding author.

## References

[1] Webb TEF Poulter M Beck J Uphill J Adamson G Campbell T. Phenotypic heterogeneity and genetic modification of P102L inherited prion disease in an international series. Brain. (2008) 131:2632–46. doi: 10.1093/brain/awn202, PMID: 18757886 PMC2570713

[2] Nozaki I Hamaguchi T Sanjo N Noguchi-Shinohara M Sakai K Nakamura Y. Prospective 10-year surveillance of human prion diseases in Japan. Brain. (2010) 133:3043–57. doi: 10.1093/brain/awq216, PMID: 20855418

[3] Ufkes NA Woodard C Dale ML. A case of Gerstmann-Straussler-Scheinker (GSS) disease with supranuclear gaze palsy. J Clin Mov Disord. (2019) 6:7. doi: 10.1186/s40734-019-0082-1, PMID: 31890235 PMC6907140

[4] Stephen CD Gusmao CM Srinivasan SR Olsen A Freua F Kok F. Gerstmann-Sträussler-Scheinker disease presenting as late-onset slowly progressive spinocerebellar ataxia, and comparative case series with neuropathology. Mov Disord Clin Pract. (2024) 11:411–23. doi: 10.1002/mdc3.13976, PMID: 38258626 PMC10982592

[5] Arata H Takashima H Hirano R Tomimitsu H Machigashira K Izumi K. Early clinical signs and imaging findings in Gerstmann-Sträussler-Scheinker syndrome (Pro102Leu). Neurology. (2006) 66:1672–8. doi: 10.1212/01.wnl.0000218211.85675.18, PMID: 16769939

[6] García C de León S Cabello JP Ortiz R Vaamonde J. Parkinsonism associated with pathological 123I-FP-CIT SPECT (DATSCAN) results as the initial manifestation of sporadic Creutzfeldt-Jakob disease. Case Rep Neurol Med. (2018) 2018:5157275. doi: 10.1155/2018/5157275, PMID: 29955403 PMC6000879

[7] Ragno M Scarcella MG Cacchiò G Capellari S Marzio FD Parchi P. Striatal [123I] FP-CIT SPECT demonstrates dopaminergic deficit in a sporadic case of Creutzfeldt-Jakob disease. Acta Neurol Scand. (2009) 119:131–4. doi: 10.1111/j.1600-0404.2008.01075.x, PMID: 18638039

[8] Akdemir ÜÖ Tokcaer AB Atay LÖ. Dopamine transporter SPECT imaging in Parkinson’s disease and parkinsoniam disorders. Turk J Med Sci. (2021) 51:400–10. doi: 10.3906/sag-2008-253, PMID: 33237660 PMC8203173

[9] Tang S Dou X Zhang Y. 18F-FP-CIT PET/CT in a case of probable sporadic Creutzfeldt-Jakob disease with parkinsonism as initial symptom. Prion. (2022) 16:91–4. doi: 10.1080/19336896.2022.2093078, PMID: 35801711 PMC9272837

[10] Jiang RY Aradi S. Gerstmann-Sträussler-Scheinker syndrome with parkinsonism, Dyskunesia, and Abnomal (I-123)-FP-CIT single-photon emission computed tomography: a case report. Cureus. (2023) 15:e50594. doi: 10.7759/cureus.50594, PMID: 38226101 PMC10788703

[11] Baiardi S Rizzi R Capellari S Bartoletti-Stella A Zangrandi A Gasparini F. Gerstmann-Sträussler-Scheinker disease (*PRNP* p. D202N) presenting with atypical parkinsonism. Neurol Genet. (2020) 6:e400. doi: 10.1212/NXG.0000000000000400 32274419 PMC7112137

[12] Umeh CC Kalakoti P Greenberg MK Notari S Cohen Y Gambetti P. Clinicopathological correlates in a *PRNP* P102L mutation carrier with rapidly progressing parkinsonism-dystonia. Mov Disord Clin Pract. (2016) 3:355–8. doi: 10.1002/mdc3.12307, PMID: 27617269 PMC5015693

[13] Tossici-Bolt L Hoffmann SM Kemp PM Mehta RL Fleming JS. Quantification of [123I] FP-CIT SPECT brain images: an accurate technique for measurement of the specific binding ratio. Eur J Nucl Med Mol Imaging. (2006) 33:1491–9. doi: 10.1007/s00259-006-0155-x, PMID: 16858570

[14] Slachevsky A Villalpando JM Satazin M Hahn-Barma V Pillon B Dubois B. Frontal assessment battery and differential diagnosis of frontotemporal dementia and Alzheimer disease. Arch Neurol. (2004) 61:1104–7. doi: 10.1001/archneur.61.7.1104 15262742

[15] Tesar A Matej R Kukal J Johanidesova S Rektorova I Vyhnalek M. Clinical Variability in P102L Gerstmann-Sträussler-Scheinker Syndrome. Ann Neurol. (2019) 86:643–52. doi: 10.1002/ana.25579, PMID: 31397917

[16] Kovács GG Puopolo M Ladogana A Pocchiari M Budka H van Duijn C. Genetic prion disease: the EUROCJD experience. Hum Genet. (2005) 118:166–74. doi: 10.1007/s00439-005-0020-1, PMID: 16187142

[17] Jiang AA Longardner K Dickson D Sell R. Gerstmann-Sträussler-Scheinker syndrome misdiagnosed as conversion disorder. BMJ Case Rep. (2019) 12:e229829:e229729. doi: 10.1136/bcr-2019-229729, PMID: 31413052 PMC6700593

[18] Shi Q Chen C Xiao K Zhou W Gao LP Chen DD. Genetic prion disease: insight from the features and experience of China national surveillance for Creutzfeldt-Jakob disease. Neurosci Bull. (2021) 37:1570–82. doi: 10.1007/s12264-021-00764-y, PMID: 34487324 PMC8566684

[19] Kang MJ Suh J An SS Kim SY Park YH. Pearls & oy-sters: challenging diagnosis of Gerstmann-Sträussler-Scheinker disease: clinical and imaging findings. Neurology. (2019) 92:101–3. doi: 10.1212/WNL.0000000000006730 30617168

[20] Higuma M Sanjo N Satoh K Shiga Y Sakai K Nozaki I. Relationships between clinicopathological features and cerebrospinal fluid biomarkers in Japanese patients with genetic prion diseases. PLoS One. (2013) 8:e60003. doi: 10.1371/journal.pone.0060003, PMID: 23555862 PMC3610658

[21] Imran M Mahmood S. An overview of human prion diseases. Virol J. (2011) 8:559. doi: 10.1186/1743-422X-8-559, PMID: 22196171 PMC3296552

[22] Rowe DB Lewis V Needham M Rodriguez M Boyd A McLean C. Novel prion protein gene mutation presenting with subacute PSP-like syndrome. Neurology. (2007) 68:868–70. doi: 10.1212/01.wnl.0000256819.61531.98, PMID: 17353478

[23] Li HF Liu ZJ Dong HL Xie JJ Zhao SY Ni W. Clinical features of Chinese patients with Gerstmann-Sträussler-Scheinker identified by targeted next-generation sequencing. Neurobiol Aging. (2017) 49:216.e1–5. doi: 10.1016/j.neurobiolaging.2016.09.018, PMID: 28340953

[24] Zhao MM Feng LS Hou S Shem PP Cui L Feng JC. Gerstmann-Sträussler-Scheinker disease: a case report. World J Clin Cases. (2019) 7:389–95. doi: 10.12998/wjcc.v7.i3.389, PMID: 30746381 PMC6369391

[25] Chen L Xu Y Fang MJ Shi YG Zhang J Zhang LL. Case report: a Chinese patient with spinocerebellar ataxia finally confirmed as Gerstmann-Sträussler-Scheinker syndrome with P102L mutation. Front Neurol. (2023) 14:1187813. doi: 10.3389/fneur.2023.1187813, PMID: 37602242 PMC10435367

[26] Karmon Y Kurzweil A Lindzen E Holmlund T Weinstock-Guttman B. Gerstmann-Sträussler-Scheinker syndrome masquerading as multiple sclerosis. J Neurol Sci. (2011) 309:55–7. doi: 10.1016/j.jns.2011.07.028, PMID: 21839476

[27] Treglia G Cason E Cortelli P Gabellini A Liguori R Bgnato A. Iodine-123 metaiodobenzylguanidine scintigraphy and iodine-123 ioflupane single photon emission computed tomography in Lewy body diseases: complementary or alternative techniques? J Neuroimaging. (2014) 24:149–54. doi: 10.1111/j.1552-6569.2012.00774.x 23163913

[28] Quintas S Samles-Falagan R Berbis MA. I^123^-FP-CIT (DATSCAN) SPECT beyond the most common causes of parkinsonism: a systematic review. Mov Disord Clin Pract. (2024) 11:613–25. doi: 10.1002/mdc3.14055, PMID: 38693679 PMC11145110

[29] Fukuoka T Nakazato Y Yamamoto M Miyake A Mitsufuji T Yamamoto T. Fatal familial insomnia initially developing parkinsonism mimicking dementia with Lewy bodies. Intern Med. (2018) 57:2719–22. doi: 10.2169/internalmedicine.0573-17, PMID: 29709939 PMC6191601

[30] Mkhitarjan T Areškevičiūtė A Lund EL Marner L Hejl AM. Sporadic fatal insomnia presenting with initial symptoms of parkinsonism and abnormal dopamine transporter imaging. Mov Disord Clin Pract. (2022) 9:249–51. doi: 10.1002/mdc3.13385, PMID: 35146064 PMC8810444

[31] Tomizawa Y Taniguchi D Furukawa Y. Genetic Creutzfeldt-Jakob disease mimicking dementia with Lewy bodies: clinical and radiological findings. J Neurol Sci. (2020) 409:116604. doi: 10.1016/j.jns.2019.116604, PMID: 31805431

[32] Vital A Fernagut PO Canron MH Joux J Bezard E Martin-Negrier ML. The nigrostriatal pathway in Creutzfeldt-Jakob disease. J Neuropathol Exp Neurol. (2009) 68:809–15. doi: 10.1097/NEN.0b013e3181abdae8, PMID: 19535991

[33] Xing XW Zhang JT Zhu F Ma L Yin D Jia W. Comparison of diffusion-weighted MRI with 18F-fluorodeoxyglucose-positron emission tomography/CT and electroencephalography in sporadic Creutzfeldt-Jakob disease. J Clin Neurosci. (2012) 19:1354–7. doi: 10.1016/j.jocn.2011.11.035, PMID: 22795494

[34] Ando S Kanazawa M Onodera O. Progressive Supranuclear palsy with predominant cerebellar ataxia. J Mov Disord. (2020) 13:20–6. doi: 10.14802/jmd.19061, PMID: 31847511 PMC6987520

[35] Costanzo M Aiello F Poleggi A Voti PL Fabbrini G Belvisi D. Progressive supranuclear palsy phenotype as an atypical clinical presentation of Creutzfeldt-Jakob disease: a case report and review of the literature. Clin Park Relat Disord. (2024) 10:100247. doi: 10.1016/j.prdoa.2024.100247, PMID: 38486940 PMC10937297

[36] Wulf MA Senatore A Aguzzi A. The biological function of the cellular prion protein: An update. BMC Biol. (2017) 15:34. doi: 10.1186/s12915-017-0375-5, PMID: 28464931 PMC5412054

[37] Fournier JG Escaig-Haye F De Villemeur TB Robain O. Ultrastructural localization of cellular prion protein (PrPc) in synaptic boutons of normal hamster hippocampus. CR Acard Sci III Adv Organ Biol. (1995) 2:99–111. 7788502

[38] Salès N Rodolfo K Hässig R Faucheux B Giamberardin LD Moya KL. Cellular prion protein localization in rodent and primate brain. Eur J Neurosci. (1998) 10:2464–71. doi: 10.1046/j.1460-9568.1998.00258.x 9749773

[39] Rial D Pamplona FA Moreira ELG Moreira KM Hipolide D Rodrigues D. Cellular prion protein is present in dopaminergic neurons and modulates the dopaminergic system. Eur J Neurosci. (2014) 40:2479–86. doi: 10.1111/ejn.12600, PMID: 24766164

[40] Herms J Tings T Gall S Madlung A Giese A Siebert H. Evidence of presynaptic location and function of the prion protein. J Neurosci. (1999) 19:8866–75. doi: 10.1523/JNEUROSCI.19-20-08866.1999, PMID: 10516306 PMC6782778

[41] Silveira JR Raymond GJ Hughson AG Race RE Sim VL Hayes SF. The most infectious prion protein particles. Nature. (2005) 437:257–61. doi: 10.1038/nature03989, PMID: 16148934 PMC1513539

[42] Bugiani O Giaccone G Piccardo P Morbin M Tagliavini F Ghetti B. Neuropathology of Gerstmann-Sträussler-Scheinker disease. Microsc Res Tech. (2000) 50:10–5. doi: 10.1002/1097-0029(20000701)50:1<10::AID-JEMT3>3.0.CO;2-6 10871543

[43] Miyaue N Tada S Ando R Iwaki H Yabe H Nishikawa N. DAT SPECT may have diagnostic value in prodromal SCA2 patients with parkinsonism. Parkinsonism Relat Disord. (2017) 44:137–41. doi: 10.1016/j.parkreldis.2017.08.012, PMID: 28844804

